# Integrating palliative care in long-term care facilities across Europe (PACE): protocol of a cluster randomized controlled trial of the ‘PACE Steps to Success’ intervention in seven countries

**DOI:** 10.1186/s12904-018-0297-1

**Published:** 2018-03-12

**Authors:** Tinne Smets, Bregje B. D. Onwuteaka-Philipsen, Rose Miranda, Lara Pivodic, Marc Tanghe, Hein van Hout, Roeline H. R. W. Pasman, Mariska Oosterveld-Vlug, Ruth Piers, Nele Van Den Noortgate, Anne B. Wichmann, Yvonne Engels, Myrra Vernooij-Dassen, Jo Hockley, Katherine Froggatt, Sheila Payne, Katarzyna Szczerbińska, Marika Kylänen, Suvi Leppäaho, Ilona Barańska, Giovanni Gambassi, Sophie Pautex, Catherine Bassal, Luc Deliens, Lieve Van den Block, on behalf of the PACE trial group, Federica Mammarella, Martina Mercuri, Paola Rossi, Ivan Segat, Agata Stodolska, Eddy Adang, Paula Andreasen, Outi Kuitunen-Kaija, Danni Collingridge Moore, Agnieszka Pac, Violetta Kijowska, Maud ten Koppel, Jenny T. van der Steen, Emilie Morgan de Paula

**Affiliations:** 1 Department of Family Medicine and Chronic Care, End-of-Life Care Research Group, Vrije Universiteit Brussel (VUB) and Ghent University, Brussels, Belgium; 2 Department of Geriatric Medicine, Ghent University Hospital, Ghent, Belgium; 3 EMGO Institute for Health and Care research, Expertise Center for Palliative Care, VU University Medical Center, Amsterdam, the Netherlands; 4 IQ healthcare, Radboud University Medical Center, Nijmegen, the Netherlands; 5 Unit for Research on Aging Society, Department of Sociology of Medicine, Epidemiology and Preventive Medicine Chair, Faculty of Medicine, Jagiellonian University Medical College, Krakow, Poland; 6 Faculty of Health Sciences, Jagiellonian University Medical College, ul. Michałowskiego 12, 31-126 Kraków, Poland; 7 National Institute for Health and Welfare, Helsinki, Finland; 8 Università Cattolica del Sacro Cuoro, Rome, Italy; 9 International Observatory on End-of-Life Care, Lancaster University, Lancaster, UK; 10 Center for the Interdisciplinary Study of Gerontology and Vulnerability (CIGEV), University of Geneva, Geneva, Switzerland; 11 Hôpitaux Universitaires de Genève, University of Geneva, Geneva, Switzerland; 12 Department of General Practice and Elderly Care Medicine, EMGO Institute for Health and Care Research, VU University Medical Center, Amsterdam, the Netherlands

**Keywords:** Nursing home, Care home, Palliative care, End-of-life care, Quality improvement

## Abstract

**Background:**

Several studies have highlighted the need for improvement in palliative care delivered to older people long-term care facilities. However, the available evidence on how to improve palliative care in these settings is weak, especially in Europe. We describe the protocol of the PACE trial aimed to 1) evaluate the effectiveness and cost-effectiveness of the ‘PACE Steps to Success’ palliative care intervention for older people in long-term care facilities, and 2) assess the implementation process and identify facilitators and barriers for implementation in different countries.

**Methods:**

We will conduct a multi-facility cluster randomised controlled trial in Belgium, Finland, Italy, the Netherlands, Poland, Switzerland and England. In total, 72 facilities will be randomized to receive the ‘Pace Steps to Success intervention’ or to ‘care as usual’. Primary outcome at resident level: quality of dying (CAD-EOLD); and at staff level: staff knowledge of palliative care (Palliative Care Survey). Secondary outcomes: resident’s quality of end-of-life care, staff self-efficacy, self-perceived educational needs, and opinions on palliative care. Economic outcomes: direct costs and quality-adjusted life years (QALYs).

Measurements are performed at baseline and after the intervention. For the resident-level outcomes, facilities report all deaths of residents in and outside the facilities over a previous four-month period and structured questionnaires are sent to (1) the administrator, (2) staff member most involved in care (3) treating general practitioner, and (4) a relative. For the staff-level outcomes, all staff who are working in the facilities are asked to complete a structured questionnaire. A process evaluation will run alongside the effectiveness evaluation in the intervention group using the RE-AIM framework.

**Discussion:**

The lack of high quality trials in palliative care has been recognized throughout the field of palliative care research. This cross-national cluster RCT designed to evaluate the impact of the palliative care intervention for long-term care facilities ‘PACE Steps to Success’ in seven countries, will provide important evidence concerning the effectiveness as well as the preconditions for optimal implementation of palliative care in nursing homes, and this within different health care systems.

**Trial registration:**

The study is registered at www.isrctn.com – ISRCTN14741671 (FP7-HEALTH-2013-INNOVATION-1 603111) Registration date: July 30, 2015.

## Background

Aging populations, rising costs and sustainable delivery of high-quality care are increasingly common concerns all over Europe [1–3]. While a growing number of older people will require palliative care in collective institutional settings, its development has only recently begun in long-term care facilities [4–7]. Long-term care facilities (in many countries labelled nursing or care homes) are collective institutional settings ‘where care – on-site provision of personal assistance with activities of daily living and on- or off-site provision of nursing and medical care – is provided for older people who live there, 24 hours a day, seven days a week, for an undefined period of time’ [6]. Although residents of such facilities are not all terminally ill, they can benefit from a palliative care approach and routine screening of palliative care needs. Initiatives supporting these facilities to integrate a palliative care approach may bring considerable added value to the sector [1, 6]. Several previous studies have highlighted the need for improvement in palliative care delivered to older people long-term care facilities [1, 5, 8].

Nevertheless, the available evidence on how to improve palliative care in these settings remains weak, particularly in Europe. A Cochrane review on the effectiveness of multi-component palliative care interventions in long-term care facilities show positive results e.g. higher satisfaction with care and fewer or shorter duration of hospital admissions [9]. Although the results are promising, these studies had several potential sources of bias and are limited to the USA [9]. In Europe, there are few studies identified, but all are small-scale and descriptive, and most were done in the Netherlands [10]. Currently, available evidence on improving the quality of palliative care in long-term care facilities, highlights the importance of a comprehensive approach in bringing about change [11–13]. Individual targeted interventions, such as training of care staff, appear ineffective if not embedded in a broader organizational approach. Rather than interventions targeting a specific element within a facility, innovative ‘complex’ palliative care interventions engaging with facilities and the wider system are needed [11–13]. Therefore, we carefully developed a complex palliative care intervention ‘PACE Steps to Success’. It aims to ensure that residents receive high-quality care in long-term care facilities in Europe through facilitating organisational change and supporting care staff to develop their roles concerning palliative care. The intervention was based on the ‘Route to Success in Long-term Care Facilities’, a palliative care intervention developed in the UK [14]. The Route to Success builds upon the well-known palliative care intervention ‘Gold Standards Framework’ (GSF), which aims to improve palliative care within primary care and was later adapted for use in long-term care facilities [15, 16].

Considering the expected future increase of older people dying in long term care facilities as recent projection studies have shown [17], there is an urgent need to increase the evidence base for delivering good palliative care in these settings. High-quality trials should evaluate the effectiveness and cost-effectiveness of palliative care, and measure effects on residents as well as staff. Since long-term care facilities across Europe vary widely in terms of type, size, ownership, or health care staff [7], it will also be important to evaluate how interventions can be implemented across a variety of different health care systems. In the PACE randomized controlled cluster trial (2014–2019), we aim to evaluate the effectiveness and cost-effectiveness of the ‘PACE Steps to Success’ palliative care intervention for older people in long-term care facilities in Europe. It will also assess the implementation process and identify facilitators and barriers for implementation across countries and in specific countries.

The aim of this article is to describe the protocol of the cluster-randomised controlled trial that will be performed to evaluate the effectiveness, implementation process, and cost-effectiveness of ‘PACE Steps to Succes’s in long-term care facilities.

## Methods

### Trial design

While a classic randomized clinical trial is regarded as the most appropriate method to study the effect of a complex intervention, it is impossible to randomise residents within a long-term care facility without contamination of the control arm [18]. For this reason, the current study is designed as a multi-facility cluster randomised controlled trial and will use pre- and post-measurement of relevant outcome variables, process evaluation and economic evaluation.

### General procedures of the cluster RCT

After inclusion, baseline data are collected in all participating facilities on 1) deceased residents (ie residents who died during the previous four months) through after-death questionnaires sent to four key respondents, 2) on staff outcomes, and 3) on facility-level characteristics. Afterwards, each facility is randomised to either the intervention or the control group, which will be done for each country separately. In the intervention group, ‘PACE Steps to Success’ will be implemented over a 12-month period. The control group will continue to provide care as usual.

After the implementation period (month 13), post-intervention data are collected on deceased residents (ie residents who died during the previous four months), staff, and facility. At month 17, post-intervention data on deceased residents (ie during the previous four months) is collected for the second time. A process evaluation will run alongside the outcome evaluation in the intervention group.

**Fig. 1:**
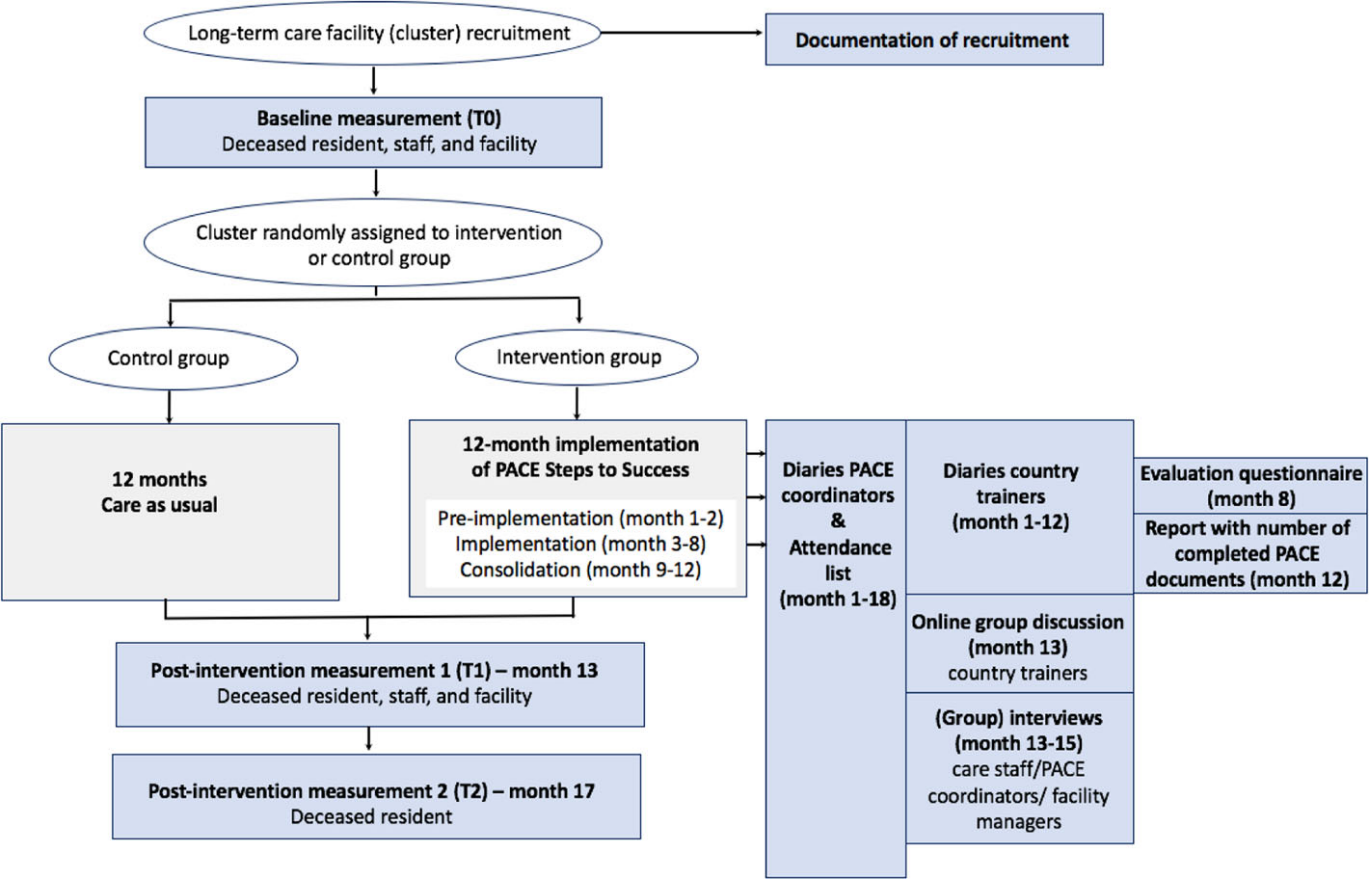
Flowchart of the cluster randomised controlled trial

The flow diagram of the study is shown in Fig. 1.

### Setting

The long-term care facilities are chosen randomly from a predefined geographical location in Belgium, the Netherlands, England, Finland, Italy, Poland, and Switzerland. These facilities have different names across countries e.g. nursing homes, care homes, homes for the elderly or residential homes [6].

*Inclusion criteria for long-term care facilities are:*

- on-site provision of nursing care and personal assistance with activities of daily living and off-site family physicians/GPs responsible for the resident’s medical care;
- Number of beds per care home at least 30;
- 15 or more residents died in or outside the nursing home over the last year (as estimated by the facilities’ managers);
- Facilties where the Board of Directors expresses explicit motivation to participate in the study and agrees to free time for a head nurse or manager to act as PACE coordinator for approximately 0.5 days per working week, depending on setting. The Board are asked to sign a letter of agreement to that effect to ensure that each LTCF remains motivated to participate, with a minimum drop-out rate.

*Exclusion criteria for long-term care facilities are:*

- Facilities already using a palliative care planning tool e.g. (accredited users of) the Gold Standards Framework, Route to Success, Six Steps to Success, interRAI-PC version or end-of-life care integrated pathways such as Liverpool Care Pathway;
- Facilities where detailed palliative care guidelines are available with corresponding high-quality practices (as judged by the researchers involved in recruitment);
- Facilties that have been involved in the development of the intervention materials.

### Study population and respondents

The Board of Directors, owner or manager in each participating facility is asked to assign one contact person for the study. The assigned contact person identifies all residents who have died over the previous four-month period and lists all care staff (nurses and care assistants) employed in the facility. To collect data on the identified deceased residents, structured questionnaires including validated instruments are sent to four key persons: administrator/manager, staff member most involved in care (preferably a nurse), treating general practitioner, and a relative (family or friend) most closely involved in patient care.

All care staff who are on duty at the time the researcher visits the facility are asked to complete a questionnaire on knowledge and attitudes towards palliative care (not linked to a particular deceased resident). For each participating long-term care facility, a facility questionnaire will be completed by the administrator/manager to collect data on facility structure, e.g. number of beds, palliative care provision.

### Recruitment of facilities

Researchers contact the facilities by phone or e-mail to organize a face-to-face meeting, to enquire whether they would be interested in participating in the study, and to evaluate inclusion and exclusion criteria. In a first telephone call, the researcher offers to send documentation introducing the project to the Board of directors/owner/manager asking for participation (voluntary-based). The documentation contains an explanation of the intervention, the full research procedure, and an agreement to participation-form to sign. The face-to-face meeting is essential to ensure motivation and dedication to participate with the intervention components and thus prevent drop out. If a facility declines to participate, another one fulfilling the inclusion criteria is selected until a sufficient number of facilities (see sample size calculation) are identified in each country. Facilities declining to participate are asked for their reason(s) not to participate.

### Randomisation

After consenting to take part in the trial, randomisation is performed. In each country, half of the facilities are randomized to the intervention group, where the ‘PACE Steps to Success’ palliative care intervention is implemented. The other half becomes the control group, where care continues as usual. Randomization is done by an independent and blinded statistician from the Netherlands. More specifically, in each country, the selected long-term care facilities are first divided into two groups: those with less than the median number of beds of the selected facilities and those with more than the median number of beds. Within each group, facilities are subsequently randomized to the intervention or the control group. The randomization procedure is repeated per country if the number of beds is unbalanced i.e. if the difference between the control and intervention groups is greater than 15%. The randomization procedure will be repeated a maximum of two times; if the unbalance persists, the last randomization result is used for the study.

### Intervention

PACE Steps to Success is a standardised palliative care intervention that aims to integrate basic or general palliative care into the day-to-day routines in long-term care facilities via a train-the-trainer programme. It is hypothesised that through the training of facility staff, staff will provide high-quality palliative care to residents, which in turn will lead to high quality of dying of residents. Table 1 shows the six steps of the intervention.

**Table 1:** The six steps of the ‘PACE Steps to Success intervention’

Steps	Tools/materials	Content of the steps
1. Discussions as the end-of-life approaches	‘Looking and Thinking Ahead’ document	Advance care planning (ACP) discussions with residents and/or families are conducted to elicit wishes and preferences around end-of-life care. This communication process usually takes place in the context of an anticipated deterioration in the individual’s condition in the future, with attendant loss of capacity to make decisions and/or ability to communicate wishes to other. ACP discussions may either be planned or ‘opportunisitic’, meaning that it can be initiated when a resident brings up the subject voluntarily. As palliative care aims to improve the quality of the remaing life, these discussions should not be just about the very last few days, but also about living well during the last years of life.
2. Assessment, care planning, and review	‘Mapping Changes in Condition’ chart	Nurses and care assistants are ideally placed to identify the various clinical triggers that indicate that a frail older person may be entering the last phase of their life. The ‘Mapping Changes in Condition’ chart plots deterioration and improvement in a resident’s physical condition. The chart helps staff recognise changes over months. By completing this every month (and every week when a resident is in the last phase of his/her life) one can see a trajectory over time of how the resident has been.
3. Co-ordination of care	Palliative Care Register Monthly multidisciplinary palliative care review meetings	Using a Palliative Care Register, residents who are identified as expected to live less than six months are discussed in detail during monthly multidisciplinary review meetings. The register prompts staff about different aspects of care to be considered. A summary sheet of those residents with particular needs is completed and sent to health professionals (such as GPs) who were not able to be present in the meeting.
4. Delivery of high-quality care	‘Long Term Care Facility Pain Assessment and Management Tool’ ‘Geriatric Depression Scale’ (short version) or ‘Cornell Depression Scale for people with dementia’	The staff is educated concerning general principles of palliative care for frail older people including those with dementia, symptom control and complex communication skills. This step also involves the training of care staff to assess and manage the particular symptoms of pain and depression. Pain assessment is undertaken on all current residents in the facility and on admission of all new residents. Assessment is continued regularly if pain is not controlled and/or at a six-monthly review. Assessment of depression is undertaken if a resident is considered depressed, or following admission when a resident settled into the nursing home if mood appears low.
5. Care in the last days of life	Integrated care plan for the last days of life	Use of an integrated care plan for the last days of life to empower staff to provide high quality care to the dying resident and their family. The Last Days of Life checklist prompts and guides the care, ensuring that appropriate medication is available or unnessary medication is discontinued in anticipation of symptoms during the dying process.
6. Care after death	Monthly reflective de-briefings groups	Monthly reflective de-briefings groups to support staff following a death and encourage experiential learning.

#### Delivery of the intervention: Train-the-trainer programme

**Fig. 2:**
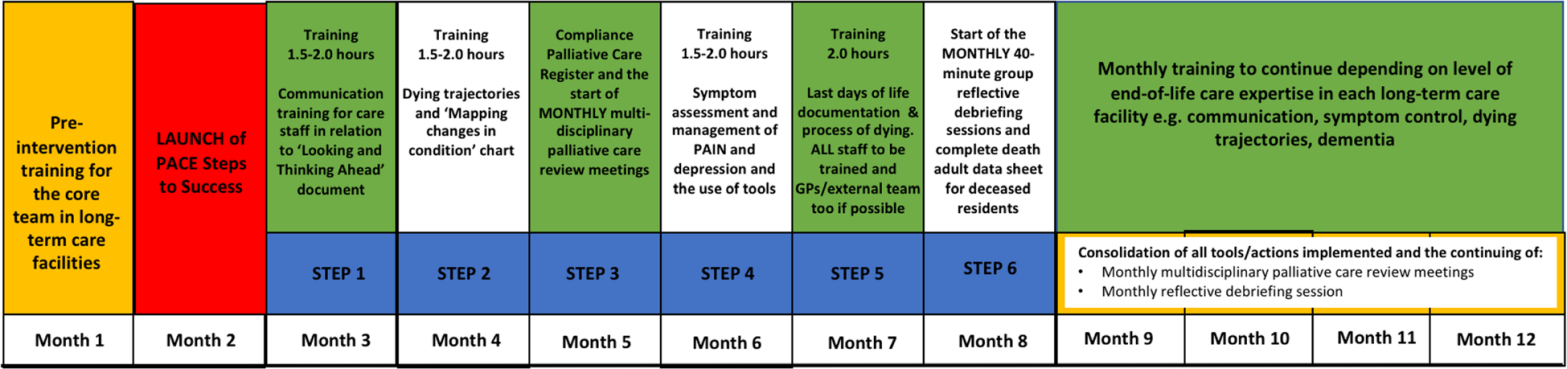
12-month implementation of PACE Steps to Success

At the core of the intervention is the nomination of a representative for palliative care within the facility (named “PACE coordinator”), ensuring that each facility has a dedicated person who has access to current national and local information. These coordinators are supported to develop their knowledge and skills and then encouraged to empower and train staff within their organization to deliver palliative care. The coordinators and staff in the facility are supported by country trainers who deliver workshops and provide support and education to all staff. The country trainers attended a two-day workshop and a follow-up meeting given by experienced international trainers (JH and KF) and are supported during the intervention study monthly by the international trainers via Google hangout sessions during the intervention. Trainers are not involved in data collection for the evaluation of the study. PACE Steps to Success will be implemented in three phases over a 12-month period (Fig. 2).

#### Development and preparation of intervention documents and materials

The intervention documents and materials (Manager and facility staff information folder, facility PACE Coordinator information folder, and a Supporting Tools folder) were first translated from English into the different country languages according to the EORTC Quality of Life Group forward-backward translation procedure [19]. Afterwards, the intervention documents and materials and the intervention implementation process were reviewed by the country trainers and by staff in several long-term care facilities in each country (moderated by the PACE country trainers, together with the researchers), that did not participate in the main trial. In a first meeting with the nursing home management, the process of review was explained and the proposed method was discussed to ensure that it was acceptable. In a second meeting with the management and at least four staff members – representing all grades and types of care staff - the ‘PACE Steps to Success’ intervention was explained and the attending staff could carefully read and comment on the materials and tools. At a third meeting with the same management and staff, the intervention materials were reviewed for ease of understanding, clarity of process, and potential barriers and facilitators to use.

Based on the findings of the review, the intervention documents and materials were adapted to make them feasible and culturally appropriate for use in long-term care facilities in the different countries.

### Outcome evaluation

#### Primary outcome

At the level of the resident, the primary outcome is quality of dying. The primary outcome at the level of the staff is staff knowledge of palliative care.

#### Secondary outcomes at staff level

Staff self-efficacy in communicating with residents at the end of life and their families, staff self-perceived educational needs regarding patients and family communication and cultural and ethical values, and staff opinions on palliative care.

#### *Secondary outcomes at resident level*

Resident’s quality of end-of-life care.

#### Economic outcomes

Direct costs and quality-adjusted life years (QALYs). Also, costs per quality increase will be calculated.

#### Other measures

Relatives’ judgement on quality of end-of-life care and quality of communication between relatives and physicians, structural facility-level characteristics, clinical and background characteristics.

The measurement instruments are described in Table 2.

**Table 2:** Overview of measurement instruments

Measurement		Unit of analysis	Respondent	Measurement instruments
Primary outcome at resident level	Quality of dying of the residents	Deceased resident	Staff	End-of-Life in Dementia Scales – Comfort Assessment while dying (EOLD-CAD) [23, 24]
Primary outcome at staff level	Staff knowledge of palliative care	Staff	Staff	Palliative care survey (PCS) construct ‘Palliative care knowledge’ [22]
Secondary outcomes	Staff self-efficacy (confidence) in communicating with residents at the end of life and their families	Staff	Staff	Self-Efficacy in End-of-Life Care Survey (S-EOLC) subscale ‘Communication’ [25]
	Staff self-perceived educational needs regarding patient and family communication and cultural and ethical values	Staff	Staff	End-of-Life Professional Caregiver Survey (EPCS), subscales ‘Patient and family communication’ and ‘cultural and ethical values’ [26]
	Staff opinions on palliative care	Staff	Staff	Rotterdam Move2PC, 11 statements regarding opinions [27]
	Quality of end-of-life care	Deceased resident	Staff	Quality of Dying in Long Term Care (QOD LTC) [28]
Economic outcomes	Resident’s health-related quality of life in last week of life in relation to direct cost of care (intervention and control)	Deceased resident	Staff	EuroQol EQ. 5D-5 L (http://www.euroqol.org/) End-of-Life in Dementia Scales – Comfort Assessment while dying (EOLD-CAD) [23, 24] Quality of Dying in Long Term Care (QOD LTC) [28]
Other measures	Quality of end-of-life care according to the relatives	Deceased resident	Relative	End-of-Life in Dementia Scales – Satisfaction with Care (EOLD-SWC) [23, 24]
	Quality of communication between relatives and physicians	Deceased resident	Relative	Family Perception of Physician-Family Communication (FPPFC) [29]
	Structural, facility level characteristics:			
	Facility status, type, case-mix, size, averaged length of stay, staffing and level of personnel	Facility	key person management	Proposal made by consortium
	Palliative care policies of facility	Facility	key person management	Based on Belgian survey [8]
	Structural quality indicators: Infrastructure, and access to palliative care	Facility	key person management	EU FP7 IMPACT Structural Quality Indicators for palliative care [30]
	Clinical and background characteristics:			
	Comorbidities and cause of death	Deceased resident	Staff GP	Based on Belgian survey [8]
	Functional and cognitive status	Deceased resident	Staff	Bedford Alzheimer Nursing Severity-Scale BANS-S [31]
	Clinical judgements on dementia and stage of dementia	Deceased resident	GP Staff	Global Deterioration Scale stage 7 (GDS) [32] Cognitive Performance Scale (CPS) [33]
	Age & gender of resident and relative, relationship to deceased	Deceased resident	Key person management Relative	Proposal made by consortium
	Timing of admission, place of death, socio-demographics, socio-economic status, religion/ethnicity	Deceased resident	Key person management Relative	Proposal made by consortium
	Age & gender of staff, experience, level of education, palliative care training	Staff	Staff	Proposal made by the consortium
	Age & gender of GP, experience, palliative care training)	Deceased resident	GP	Proposal made by the consortium

### Process evaluation

Alongside the outcome evaluation, we will undertake an embedded process evaluation using the RE-AIM framework. This framework structures the different implementation factors that are considered important for implementation effectiveness, namely Reach, Efficacy, Adoption, Implementation, and Maintenance [20]. An overview of the measures used in the process evaluation can be seen in Table 3.

**Table 3:** Operationalization of RE-AIM dimensions measurement methods

Dimension	Operationalized in pace process evaluation	Measurement methods
Reach *(Proportion of caregivers in care settings that participated in the intervention during the study)*	- Number of participants (care staff attending each training or meeting) divided by the total number of care staff (eligible participants) who work in the facility or facility unit - Comparing characteristics of participating facilities with non-participating facilities	- Attendance lists of trainings and meetings - Documentation of recruitment process by the researcher
Efficacy *(primary and secondary outcomes (positive and negative)*	- Primary and secondary outcome measures	- Questionnaires about deceased resident - Questionnaire about staff (see Table 2)
Adoption *(Extent to which caregivers actually adopt the intervention in the study (followed the intervention or showed compliance with the intervention)*	- Number of Looking and Thinking Ahead Forms documented and Pain/Depression Assessments documented - Experiences with applying the intervention steps in daily practice (e.g. reasons for (not) applying steps, changes in practice)	- Report from PACE coordinators - Group interviews with care staff and PACE coordinators
Implementation *(Extent to which the intervention is implemented as intended in the real world, including implementation barriers and facilitators)*	- Fidelity: extent to which the steps of the intervention were delivered as intended (frequency, order and content of the sessions) - Satisfaction of care staff members towards the intervention program and trainer’s competences - Barriers and facilitators for implementation	- Structured diaries filled in by country trainers - Evaluation questionnaire after last training session filled in by care staff - List of Barriers and Facilitators for Implementation, added to the Nurses’ experiences and attitudes questionnaire - Group interviews with care staff and PACE coordinators - Online discussion groups with trainers from all countries - Semi-structured interviews with nursing home managers - Structured diaries for country trainers and PACE coordinators
(Intention to) Maintenance *(Extent to which the intervention is intended to be sustained over time)* ^a^	- Care staff members’ intention for using PACE documents in the future - Organizational intention for long-term implementation - Recommendations for improving usability of intervention program	- Evaluation questionnaire after last training session - Semi-structured interviews with facility managers - Group interviews with care staff - Group interview with PACE coordinators - Online discussion group with trainers from all countries

^a^Because of the limited duration of the study, we will measure intention for maintenance instead of actual maintenance

### Sample size calculation

The primary outcome at resident level was used to estimate the sample size required to detect significant differences between intervention and control. The power calculation is aimed at comparing the intervention group with the control group across all countries, taking the multilevel nature of the data into account. Assuming coefficient of variation of true means between clusters/countries within each group of 0.09, group sample sizes of 144 (4 deaths each in 36 LTCFs per study arm across countries) achieve 90% power to detect a difference of − 3 in quality of dying (CAD-EOLD scale) and α = .05. CAD-EOLD scores are based on a comparative Belgian-Dutch study [21].

Taking into account a nonresponse of 20% for staff and 50% for relatives, we would need to include a minimum of 576 deaths to ensure we can achieve the power as described above. We estimate that an average number of 5–6 deaths that can be be identified over a four-month measurement period per facility of more than 30 beds. A multi-country database of 72 facilities will be constructed, for an estimated 432 deaths per four-month period. By measuring over two such periods, we expect a total of 864 deaths (432 of those with relative responses), leaving enough room to compensate for lower mortality rates.

### Analyses

All data collected through the different questionnaires will be encoded and stored in Limesurvey. Data cleaning will be carried out using IBM SPSS syntax operations. All analyses will be two-tailed with 95% confidence intervals (CIs) and considered significant if α < 0.05.

#### Outcome and cost evaluation analyses

Anova (normal distribution) or Mann-Whitney U-test (non-normal distribution) for continuous, and χ^2^ tests for categorical variables will be used to describe differences between the control group and the intervention group in both the baseline and post-intervention measurements and for post-hoc non-response analyses. Cluster and study population characteristics will be reported as mean and standard deviation (SD) for continuous variables or frequency and percentage for categorical variables.

We will analyse differences for the primary and secondary outcomes between the control and the intervention group using multilevel mixed model analyses that will account for the baseline measurement and the clustered study design (i.e. residents and staff nested within a facility or country). Outcomes will be analyzed with facility and country as random factor, and group, time point, and their interaction as fixed factors. We will calculate differences in mean change (post-intervention measurements minus baseline) between the intervention group and the control group (interaction group*time). Results will be expressed as estimated means with corresponding 95% CIs. Comparisons will be reported in terms of expected mean differences with 95% CIs. To interpret the magnitude of the effects for the different outcomes, we will estimate effect sizes (Cohen’s *d*) using the baseline-adjusted mean differences and the variance between residents, between facilities, and between country.(20) The primary statistical analyses will be by intention-to-treat. All analyses will be performed using a statistical software that supports multi-level mixed model analyses – e.g. IBM SPSS Statistics 24: Release 24 (IBM Corporation) or STATA.

Differences in costs and effects will be analyzed by means of a generalized linear model (GLM), with long term care facility as cluster. Costs are often right skewed since there are no negative costs and some patients incur high costs. Therefore, a gamma distribution will be used. Moreover, a log link will be included as a linear relation is not assumed. To analyze cost-effectiveness, the net monetary benefit (NMB) parameter will be calculated. Then the NMB will be regressed on a set of independent variables such as group (intervention vs. control), facility (cluster) and potential confounders such as disease severity. Means and 95% confidence intervals will be presented. Finally, the results are presented by means of a cost-effectiveness acceptability curve (CEAC) that is able to evaluate the probability being cost-effective by using different WTP thresholds for an extra QALY.

#### Proces evaluation analysis

Based on key elements of the PACE intervention, criteria for a high, medium and low level of Reach, Adoption, Implementation and intention for Maintenance will be defined. For example, Reach will be rated as ‘high’ if the mean attendance rate on all six training sessions/meetings is 70% or higher, ‘medium’ if 30–69% and ‘low’ if below 30%. Using descriptive statistics and other outcomes that will be gathered with the measurement methods described in Table 1, we will display how each long-term care facility within each country performs on the different dimensions. In addition, we will combine the ratings for Reach, Adoption and Implementation into a combined PACE RAI-score, which can be linked to the primary and secondary outcomes of the PACE study; e.g. to analyze whether a better Reach, Adoption and Implementation of the PACE Program results in better effect outcomes. The PACE RAI-score ranges from 0 (if Reach, Adoption and Implementation are all rated as ‘low’) to 6 (if Reach, Adoption and Implementation are all rated as ‘high’).

The qualitative data that will be gathered in the online discussion groups and (group) interviews will be analyzed according to the principles of thematic analysis, in a more deductive way (i.e. framework approach). In each country, two researchers will read and reread the transcripts of the (group) interviews in their native language to become thoroughly familiar with the data. They will write extensive summaries including illustrative quotes in English, facilitated by templates in which themes are already pre-structured to some extent. Analysis of the cross-country data described in these summaries will be done by two researchers from VUmc, and then discussed with different members of the research team from all countries, in order to work towards a consensus about interpretation of the key findings.

### Informed consent

All persons participating in the study (facility managers, care staff, GPs, relatives, and the concerned residents and relatives in the interviews for the process evaluation) have to give their prior informed consent in writing. If residents are unable to give informed consent, they will not be involved in the study. In some countries, such as Poland and the Netherlands, a separate informed consent is not required if questionnaires are filled in anonymously.

### Ethics approval

Ethics approval from the relevant ethics committees were obtained in all participating countries. Belgium: Commissie Medische Ethiek UZBrussel, 27/05/2015; England: NHS – NRES Committee North West-Haydock, 10/09/2015; Finland: Terveyden jahyvinvoinnin laitos, Institutet för hälsa och välfärd, 30/6/2015; Italy: Comitato Etico, Universita Cattolica del Sacro Cuore, 6/11/2017; Netherlands: Medisch Ethische Toetsingscommissie VUMedisch Centrum, 2/7/2015; Poland: Komisja Bioetycza, Uniwersytetu Jagiellonskiego, 25/6/2015; Switzerland: Commission cantonale d’éthique de la recherché scientifique de Genève (CCER), 6/8/2015.

## Discussion

The lack of high quality trials in palliative care has been recognized throughout the whole field of palliative care research [1, 5, 10]. This cluster randomized controlled trial, designed to evaluate the impact of the palliative care intervention for long-term care facilities ‘PACE Steps to Success’ in seven countries, is unprecedented and will provide important evidence concerning the effectiveness, implementation, and cost-effectiveness of palliative care for residents of nursing homes.

Unique in the PACE trial is the cross-country set-up with the participating countries having distinct health and long-term care systems. The trial’s strengths also include the focus on outcome and costs, in combination with an in-depth process evaluation, studying the degree of implementation of the intervention. The embedded process evaluation is important in providing evidence on barriers and facilitators for palliative care implementation in practice and on relevant contextual factors in successfully implementing such complex interventions. It will ensure that the findings of the trial are informative to practice and policy regardless of whether evidence is found for an effect on the primary outcomes. The strengths of the study furthermore lie in its pragmatic nature, measuring effectiveness of the intervention in routine nursing home practice, which will enhance the external validity of the findings. The intervention materials are available in six languages (English, Dutch, Finnish, French, Italian, and Polish) and are adapted to the different cultural contexts, adding further to the external validity of the study.

The intervention used in this study is carefully chosen and based on the available evidence of what works best to bring about change in long-term care facilities. When implementing an innovative and complex intervention in these facilities it is important to take into account the facility context, such as leadership, culture, staff capacity to engage with an innovation, and existing patterns of working and communicating, as it greatly influences how long-term care facilities deal with innovations and their implementation [12]. In the ‘PACE Steps to Success’ intervention, we took into account country and context-specific elements as much as possible during the intervention development and testing phase, and the embedded process evaluation using the RE-AIM framework will give insight into the general and country-specific facilitators and barriers for implementation of the innovative and complex intervention in long-term care facilities.

There are also limitations to the study. First, because the trial is powered across the participating countries, we will not be able to assess the effectiveness of the intervention in a particular country. Second, there is uncertainty whether the Palliative Care Survey instrument [22] used to measure the primary outcome of the trial on the staff level, is sensitive enough to detect changes in staff knowledge over a 1-year time period. Third, we will do the post-intervention evaluation of the quality of dying (using the CAD-EOLD instrument [23, 24]) of residents who have died between month 9 and month 17, while the intervention runs from month 1 tot 12. Hence, some residents will have died before the intervention is fully delivered, and others when it has been finalised for some months already. Thus, it mightbe difficult to truly capture the effects of the intervention. Forth, due to the nature of the study design and the intervention, blinding of treatment allocation is not possible. The nurses and care assistants who fill in the questionnaires are aware of and are trained in delivering the intervention, which might affect their responses on the outcome measures (i.e. detection or ascertainment bias). However, in view of the need for evaluations related to the end of life of the nursing home residents of key persons involved in care such as nurses and care assistants, we deem their assessments of the primary outcome at the resident level an appropriate choice for the study.

## Conclusion

The cross-national cluster RCT of the PACE project will be the first trial aimed at measuring the effectiveness and cost-effectiveness of the intervention ‘PACE Steps to Success’ to improve palliative care for residents in long-term care facilities within different health care systems and on a wide range of outcomes for staff and residents. Combined with costs and an in-depth process evaluation, this study will add considerably to the evidence on the implementation of palliative care for residents of long-term care facilities in different countries. Considering the expected large increase of elderly people needing institutional care at the end of life, the ‘PACE Steps to Success’ trial is urgently needed.
